# Automated serial electron diffraction: implementation in *LibraEDT* and its applications

**DOI:** 10.1107/S1600576726003894

**Published:** 2026-05-08

**Authors:** Moussa D. Faye Diouf, Danilo Marchetti, Paola Parlanti, Alessandro Pedrini, Mauro Gemmi

**Affiliations:** a Electron Crystallography, Istituto Italiano di Tecnologia, Pontedera 56025, Italy; b University of Parma – Department of Chemical, Life and Environmental Sustainability Sciences, University of Parma, Parma 43123, Italy; SLAC National Accelerator Laboratory, Menlo Park, USA

**Keywords:** 3D electron diffraction, 3D-ED, serial electron diffraction, SerialED, serial precession electron diffraction, *LibraEDT*

## Abstract

Serial electron diffraction (SerialED) offers an effective route for structure determination of beam-sensitive materials, yet its adoption has been limited by the practical challenges of manual acquisition and the handling of large datasets. In this work, we introduce the implementation of continuous SerialED, also known as stage-scanning SerialED, within *LibraEDT* for unsupervised data collection. To illustrate its practicality, SerialED datasets were collected with and without precession and later processed with minimal additional effort, showing that the acquisition strategy is compatible with standard data processing tools.

## Abstract 2

We describe a continuous serial electron diffraction implementation in *LibraEDT* that enables automated data collection with real-time frame evaluation, together with an accessible workflow for subsequent data processing.

## 1. Introduction

Serial crystallography (SX), a technique originally developed for X-ray diffraction (Chapman *et al.*, 2011 ▸), has transformed the structural analysis of sensitive and complex materials. Central to this approach is the principle of diffraction before destruction (Chapman *et al.*, 2014 ▸), which allows data collection from a crystal before it suffers significant damage from the radiation dose. This concept is particularly relevant for biological macromolecules and many other materials that degrade rapidly under intense radiation exposure.

X-ray serial crystallography uses two main methods for data collection: liquid-jet injectors and fixed-target setups. Liquid-jet injectors work by suspending crystals in a liquid or viscous medium and delivering them into the X-ray beam path for continuous exposure to fresh crystals during data collection. Alternatively, fixed-target setups immobilize crystals on specialized substrates, such as silicon wafers or microfabricated grids, allowing for systematic scanning of the sample. In both methods, diffraction data are collected from different and randomly oriented crystals in single shots, allowing for the reconstruction of a complete dataset even when individual crystals are too small or too sensitive for traditional crystallographic techniques. While these tech­niques have proven to be successful in structural analysis, they rely on specialized infrastructure such as synchrotron radiation facilities or X-ray free-electron lasers, which are costly to operate and have limited accessibility.

Transmission electron microscopes (TEMs), thanks to their versatility, naturally provide all the essential tools to perform serial electron crystallography. Widely available in most research laboratories, TEMs are equipped with a controllable electron beam and a movable stage, enabling precise targeting of specific areas of interest. Sample preparation is simple and can be adapted to make sure that densely packed nanocrystals are distributed across the grid. The strong interaction between electrons and the specimen (Vajnštejn, 1956 ▸; Bethe, 1928 ▸), along with the ability to adjust the beam size to just a few nanometres, results in data with high signal-to-noise ratios. Furthermore, transition between imaging and diffraction modes is relatively quick, making data acquisition much easier. This makes serial electron diffraction (SerialED), which is based on the same principle of diffraction before destruction, ideal for structure determination of a wide range of beam-sensitive materials, such as metal–organic frameworks (MOFs) (Wang *et al.*, 2019 ▸), hybrid materials, organic crystals (Pulleri Vadhyar *et al.*, 2025 ▸) and macromolecular crystals (Bücker *et al.*, 2020 ▸), that would otherwise be amorphized by the electron beam or degraded under the high-vacuum conditions of the TEM column (below 10^−7^ mbar).

SerialED data acquisition can be performed in different ways, with the most common approaches being either by shifting the electron beam across the different regions of the grid (Bücker *et al.*, 2021 ▸) or stage scanning, where the beam is kept fixed and the stage is moved instead, similarly to fixed-target SX (Hofer *et al.*, 2025 ▸). Beam shifting is generally faster as it benefits from the fast response of beam deflectors, but it introduces shifts in the central diffraction beam and parabolic distortions (Brázda *et al.*, 2022 ▸) that may require post-acquisition corrections depending on the data processing tools used. Stage scanning, by contrast, is slower but makes sure that diffraction conditions remain stable over a large area of the grid. Both methods produce large datasets, often containing many non-diffracting or low-quality frames, which must be filtered using robust frame selection algorithms.

To improve targeting efficiency, low-magnification images can be taken to identify promising areas for diffraction data collection (Cichocka *et al.*, 2018 ▸; Hogan-Lamarre *et al.*, 2024 ▸). More advanced strategies involve the use of image recognition algorithms, which are actively being developed to increase the speed and robustness of crystal recognition. These tools can automate crystal selection and direct either beam or stage shifts for targeted acquisition. Finally, SerialED can also be performed without prior imaging (‘blind scanning’). In this case the TEM grid is systematically scanned through stage or beam movements, minimizing pre-exposure and the associated radiation damage.

Despite its numerous advantages, SerialED has been reported in only a few published articles for structure determination (Plana-Ruiz *et al.*, 2025 ▸; Hofer *et al.*, 2025 ▸; Hogan-Lamarre *et al.*, 2024 ▸; Bücker *et al.*, 2021 ▸; Smeets *et al.*, 2018 ▸). This is largely due to the tedious nature of manual data acquisition (which can require measuring hundreds or even thousands of crystals depending on the symmetry), the time-consuming data processing and the fact that, at the time of writing, prior knowledge of the unit-cell parameters is still required.

In this work, we present the implementation of continuous SerialED (Hofer *et al.*, 2025 ▸) in the *LibraEDT* software (Faye Diouf & Gemmi, 2025 ▸) and discuss data processing using two different software applications, *nXDS* (Kabsch, 2014 ▸) and *PETS2* (Palatinus *et al.*, 2019 ▸). We demonstrate the method on the beam-sensitive MOF MIL-125(Ti) (Dan-Hardi *et al.*, 2009 ▸), a titanium-based framework that rapidly degrades under the electron beam and is therefore challenging to study using conventional 3D electron diffraction (3D-ED) (Gemmi *et al.*, 2019 ▸). While the structure of MIL-125(Ti) is already known, the only reported structural model was obtained from powder X-ray diffraction (PXRD) and suffers from imperfections in the geometry of the organic linker.

## 2. Implementation and features

For unsupervised SerialED data acquisition, control of the microscope and detector hardware is essential. *LibraEDT* is software developed for ED data collection for the Zeiss Libra 120 TEM. *LibraEDT* directly communicates with the TEM and the ASI Timepix detector, allowing control of several components such as the electron beam, which can be shifted via the microscope coils, the stage, which can be moved and rotated, and the detector, which can be triggered for image or diffraction pattern acquisition. A detailed description of the overall *LibraEDT* framework and additional features such as crystal tracking for 3D-ED has been provided in a previous publication (Faye Diouf & Gemmi, 2025 ▸). In the present work, we expand on this implementation by focusing on the SerialED capabilities (highlighted in Panel 5 of Fig. S1), which allow for unsupervised acquisition of high-quality diffraction data.

### 2.1. Challenges and parameter optimization

#### 2.1.1. Effect of crystal size

**Figure 1:**
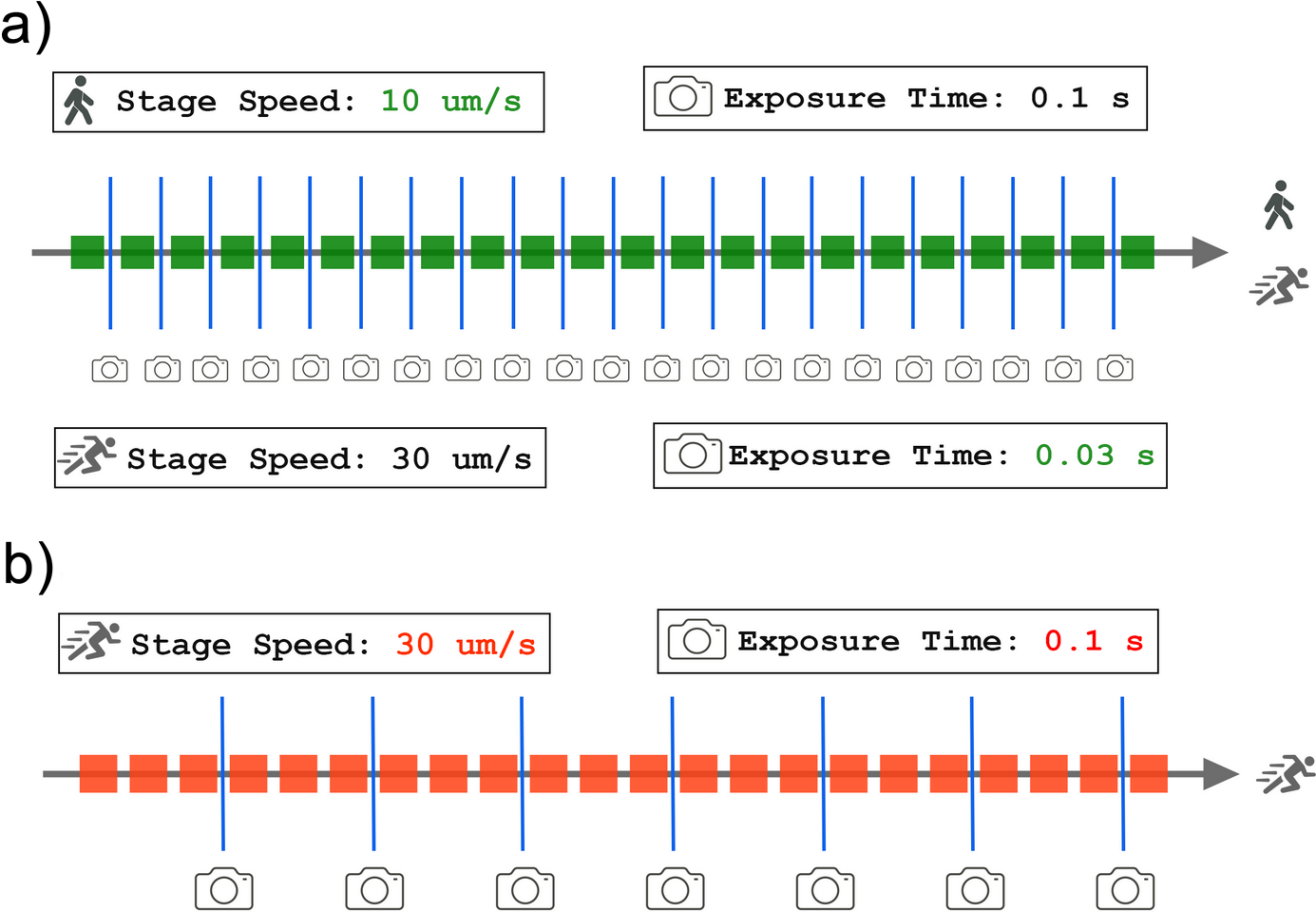
Effect of stage displacement per frame on the number of crystals illuminated per diffraction frame. Red and green cubes represent individual 1 × 1 µm crystals. (*a*) When the displacement per frame (1 µm) is comparable to the crystal size, each diffraction frame primarily corresponds to a single crystal. This condition can be achieved either by reducing the stage speed (top) or by shortening the exposure time (bottom). (*b*) When the stage displacement per frame (3 µm) exceeds the crystal size, multiple crystals are illuminated simultaneously, leading to overlapping diffraction.

Successful SerialED acquisition by stage scanning requires careful tuning of the experimental parameters to make sure that most diffraction frames primarily correspond to single crystals, avoiding multiple crystals diffracting at the same time inside the illuminated area. Since in our SerialED implementation the patterns are collected while the stage is continuously moving, a critical parameter to avoid crystal superposition is the stage displacement per frame or scanning resolution (in µm), defined as the product of the stage speed (in µm s^−1^) and the exposure time (in s). Ideally, this stage displacement per frame should be comparable to the crystal dimensions [Fig. 1 ▸(*a*)]. Smaller displacements will result in consecutive frames containing redundant diffraction for the same crystal, while excessively large displacements per frame increase the probability of recording patterns from multiple crystallites [Fig. 1 ▸(*b*)]. Achieving the optimal balance between these parameters is essential for maintaining both data quality and acquisition efficiency. In general, larger crystals allow for faster stage movement and longer exposure times, while smaller crystals require shorter exposures and slower stage movement to minimize diffraction overlap between adjacent particles. Also, decreasing the stage speed significantly extends the total acquisition time and increases the electron fluence per crystal, since each point spends more time under the beam. Reducing the exposure time, on the other hand, does not directly affect the fluence but produces more frames with weaker diffraction signal, which in practice may require an increase in beam current to maintain sufficient intensity. Finding the right balance between these parameters is therefore essential, especially when working with beam-sensitive materials. From our experience, SerialED data acquisition using this approach is much easier to perform on larger crystals ( >1 µm) which allow for more flexibility in the choice of experimental parameters.

#### 2.1.2. Precession cycles

As recently reported (Plana-Ruiz *et al.*, 2025 ▸) SerialED can benefit from collecting the patterns using precession (Vincent & Midgley, 1994 ▸). In fact, in precession mode, every reflection is integrated over the excitation error, making the recorded intensity less dependent on the crystal orientation. There are no limitations in using *LibraEDT* with precession, provided some additional constraints are considered in selecting the appropriate exposure time. When SerialED is performed with precession, the frequency of the precession movement (100 Hz in our setup) should be taken into consideration. If the exposure time of the detector is shorter or it is not a multiple of the precession period (in our setup 10 ms), the diffraction pattern will correspond to an incomplete precession cycle, leading to non-uniform intensity averaging. Therefore, the exposure time should be chosen to be an integer multiple of the precession period or long enough to include several full cycles, while still keeping the displacement per frame within an appropriate range.

#### 2.1.3. Frame selection in real time

This SerialED approach generates a substantial volume of data, particularly when working with small crystals, where shorter exposure times are typically required. Modern single-electron detectors can record several thousand frames per second (fps), quickly producing datasets that can reach hundreds to thousands of gigabytes (GB) within a single experiment. To manage the large amount of data generated and only retain high-quality diffraction frames, *LibraEDT* performs real-time processing of each frame using the *Cheetah* PeakFinder8 algorithm (Barty *et al.*, 2014 ▸), originally developed for serial X-ray crystallography and also implemented within *CrystFEL* (White *et al.*, 2012 ▸). In this implementation, every acquired diffraction pattern is immediately analysed, and only those meeting user-defined criteria are saved to disk. For tuning the SerialED experiment, the user can set the following parameters within the software’s GUI (Fig. S1):

(1) *d_max*: defines the minimum resolution (in Å^−1^) considered for peak detection, ignoring reflections below the specified value.

(2) *Threshold*: specifies the minimum pixel intensity required for a signal to be classified as a diffraction peak.

(3) *Peaks*: sets the minimum number of peaks that must be detected in a frame for it to be accepted.

(4) *Peak Size*: defines the expected spot size (in pixels) for peak detection.

(5) *I/Sig*: establishes the minimum signal-to-noise ratio required for the detected peaks.

**Figure 2:**
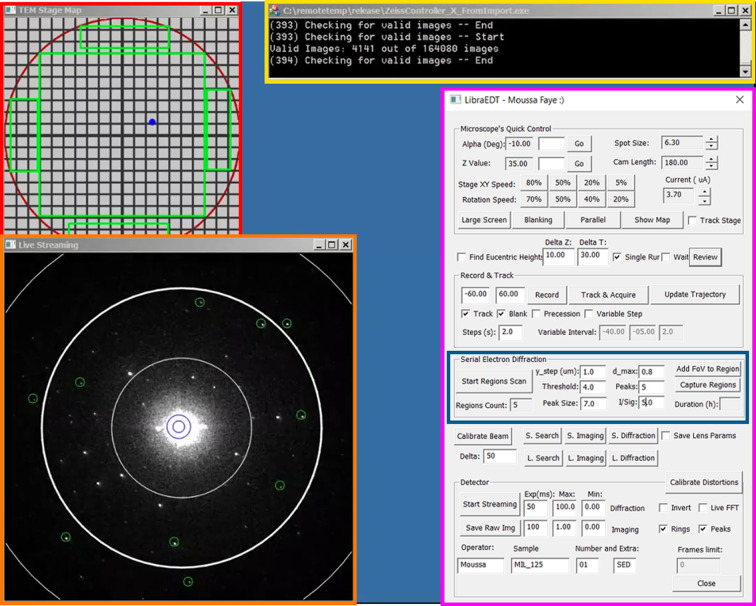
The *LibraEDT* interface during data acquisition. The red box highlights the interactive 2D stage map, showing the grid layout and regions marked for scanning. The yellow box displays the log window, tracking the progress of image acquisition and filtering. The pink box shows *LibraEDT*’s GUI and data acquisition parameters. The blue box focuses on the *Serial Electron Diffraction* settings, where step size, thresholds and filtering conditions are configured. Lastly, the orange box shows the live feed of diffraction patterns, with resolution rings and detected peaks based on the previously set conditions.

All of the parameters used for the filtering of the diffraction frames can be changed at any time during data acquisition within *LibraEDT*’s GUI (Fig. S1). This flexibility provides the ability to apply stricter or more relaxed filtering criteria as needed depending on the quality of the crystals. For instance, if many valuable frames are being excluded, the resolution limit can be adjusted to include lower-resolution reflections, or the thresholds for the minimum number of reflections and their intensity-to-noise ratio can be relaxed accordingly. The main metrics manually monitored by the user during acquisition are the number of accepted frames relative to the total number of processed frames and the live visualization of detected peaks overlaid on each diffraction pattern (Fig. 2).

#### 2.1.4. Peak processing speed and stability

Peak processing algorithms are computationally expensive. Therefore, *LibraEDT* must carefully optimize the computing resources to be able to process diffraction frames in real time, avoiding memory build-up or system crashes. To achieve this, the acquired images are first binned. Then, by adopting the user-defined *d_max* value, a mask is applied around the central beam, whose position is determined by a dedicated detection routine. Only pixels outside the mask are considered for peak detection. These steps significantly reduce the number of pixels to be checked, consequently decreasing the computational load. To further improve throughput, *LibraEDT* processes frames in batches of 1000 using multithreading, allowing simultaneous processing of multiple frames without interrupting data acquisition. However, for safety, the software continuously monitors memory usage and temporarily pauses acquisition if the processing queue grows faster than the system can handle. The queued frames are then processed before acquisition resumes. During our implementation, this algorithm has been successfully tested with a minimum exposure time of 10 ms (100 fps) without any crashes or interruptions in data acquisition.

### 2.2. Experimental setup

The experiments discussed in this work were carried out on a Zeiss Libra 120 TEM equipped with an in-column Omega filter, an ASI Timepix single-electron detector and a NanoMEGAS DigiSTAR P1000 precession system operating at 100 Hz. Within the Zeiss Libra TEM, the user is able to freely change the stage speed from 5 to 100 µm s^−1^.

### 2.3. Preparation for data acquisition

In *LibraEDT*, SerialED data acquisition is performed by systematically shifting the stage while keeping the beam fixed. This approach is analogous to the fixed-target method in SX (Hunter *et al.*, 2014 ▸; Jaho *et al.*, 2024 ▸) and was first proposed by Hofer *et al.* (2025 ▸). Within a given scan area (referred to as a *Region* in *LibraEDT*), the stage follows a serpentine path, sweeping from left to right across one line, and then shifting down to sweep back from right to left and so on. The vertical step size for this downward shift can be defined directly in the GUI by adjusting the *y_step* value (in µm).

There are two ways to define regions in *LibraEDT*. The *Show Map* button (Panel 1 in Fig. S1) can be used to display an interactive 2D map of the grid, on which the user can draw one or multiple square regions to be scanned (Fig. S2). Alternatively, regions can be defined through the *Add FoV to Region* button (where FOV denotes field of view), which automatically creates a scan region on the map based on the current stage coordinates, the calibrated pixel size and the dimensions of the acquisition window. The first method is particularly useful for covering large parts of the grid or multiple distant areas without pre-exposing the sample, while the second provides a convenient way to target densely packed areas identified at low or normal magnification.

### 2.4. Data acquisition workflow

An overview of the *LibraEDT* interface during SerialED data acquisition is shown in Fig. 2 ▸. The different windows displayed in the software provide full control over the microscope, stage and detector, allowing for a completely automated workflow.

**Figure 3:**
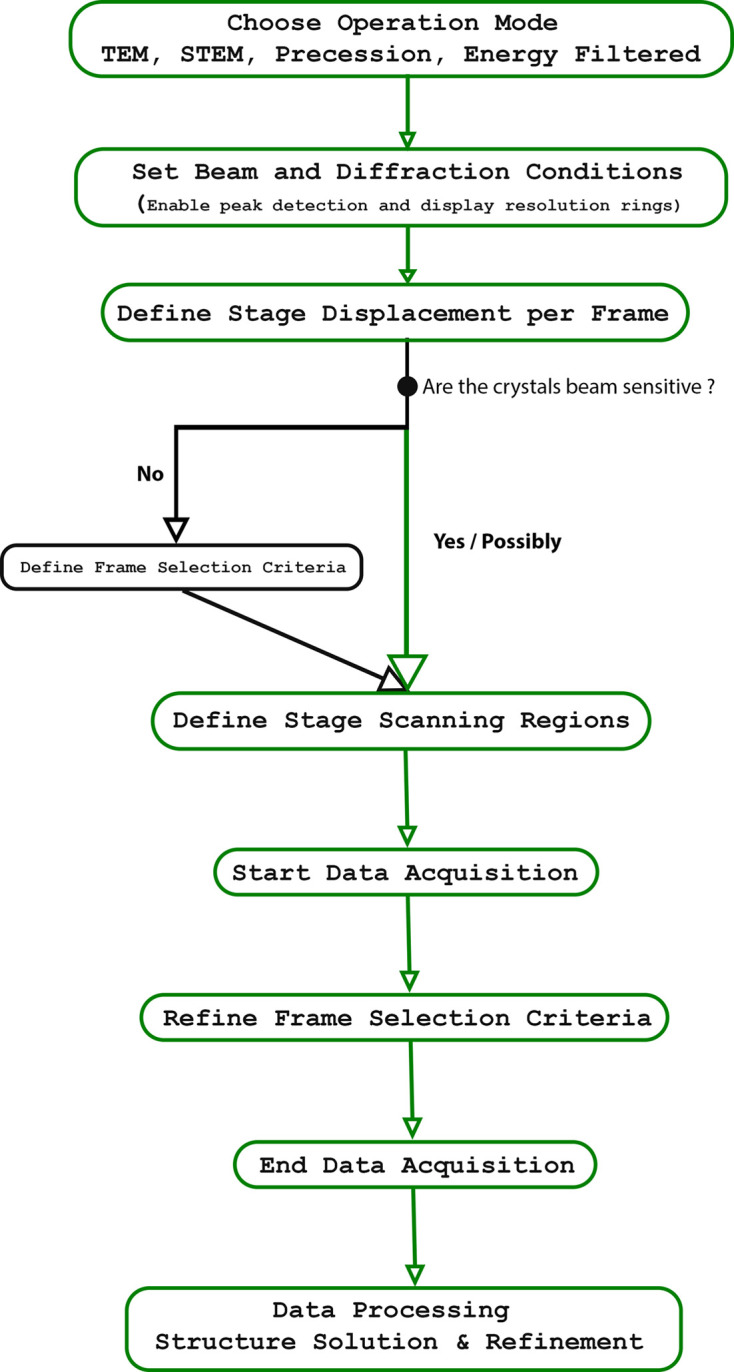
Workflow for SerialED data acquisition using *LibraEDT*.

The process begins by selecting the operation mode [TEM or scanning TEM (STEM)] with optional precession and/or energy filtering. This is followed by the setup of beam and diffraction conditions while enabling peak detection and displaying resolution rings. The stage displacement per frame is then defined by adjusting the combination of stage speed and exposure time, as described in Section 2.1.1. If the material is not beam sensitive, diffraction patterns of some crystals can be displayed in real time to establish suitable frame selection criteria (the minimum *I*/σ, number of peaks and resolution threshold). Once the conditions are optimized, the user defines one or more scan regions, as detailed in Section 2.3, after which SerialED acquisition can be started. During data acquisition, the filtering parameters can be refined (or defined if not done previously) at any moment on the basis of the crystal quality. After acquisition, the retained diffraction frames are corrected (cross-correction, dead-pixel correction, flat-field correction *etc*.) in preparation for data processing. The complete SerialED data acquisition workflow, summarizing these steps, is illustrated schematically in Fig. 3 ▸.

## 3. Application and data analysis

### 3.1. Sample preparation and data acquisition

MIL-125(Ti) was synthesized via a modified version of a previously reported procedure (Sun *et al.*, 2024 ▸). A concentrated solution of the material was deposited onto a holey carbon TEM grid, resulting in a dense distribution of nanocrystals across the grid surface.

Data acquisition was carried out using *LibraEDT* without the need for prior low-magnification imaging. Instead, the interactive *2D Map* feature was used to directly select regions for scanning. Two different data collections were carried out: one with steady parallel beam (we will refer to it simply as SED) and a second one in precession electron diffraction mode (we will refer to it as SPED, serial precession ED). The data collection parameters are summarized in Table 1 ▸.

**Figure 4:**
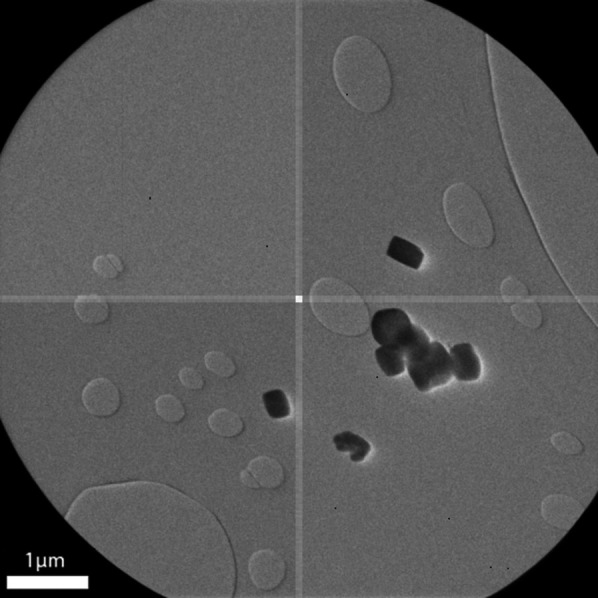
TEM image showing the morphology of the MIL-125(Ti) crystals. The size of the crystals ranges from 200 to 400 nm, as indicated by the 1 µm scale bar.

In SED, as a measure against potential preferred orientation, the stage was randomly rotated every 45 min by an angle within a ±35° interval. The acquisition then proceeded in an unsupervised manner overnight, running for approximately 15 h. The stage was set to move at a constant speed of 10 µm s^−1^ and diffraction patterns were recorded every 50 ms. This resulted in a stage displacement per frame of 0.5 µm (500 nm), which is comparable to the size of the crystals (Fig. 4 ▸). Given the beam size and exposure time, the resulting electron fluence per crystal is approximately 12 e^−^ Å^−2^. This is higher than the fluence typically used in continuous-rotation electron diffraction experiments, which is usually below 1 e^−^ Å^−2^. However, since each crystal is exposed only once, radiation damage remains limited.

**Figure 5:**
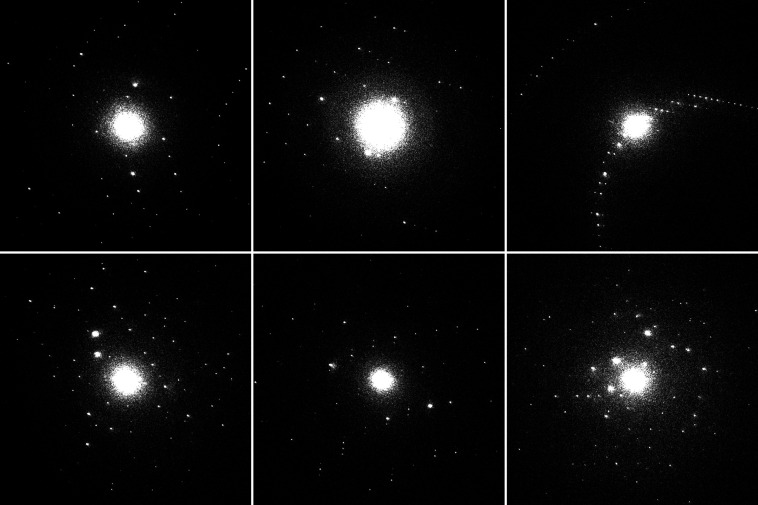
Six randomly selected images from the retained frames of the SED dataset, all of which met the filtering criteria [resolution better than 0.80 Å^−1^ (1.25 Å), at least 5 peaks with *I*/σ > 5]. Note that the selection includes images containing diffraction patterns from multiple crystals.

The data acquisition was stopped the next day with over 1000000 images (∼500 GB) collected. However, not all of these images were saved to disk as they were being peak-processed in real time using the filtering criteria defined in the GUI. Therefore only frames containing at least five diffraction peaks with an intensity-to-noise ratio (*I*/σ) exceeding 5 and a resolution for each peak exceeding 0.80 Å^−1^ (1.25 Å) were retained, resulting in a final dataset of 26962 high-quality frames (∼13.5 GB) suitable for data processing (Fig. 5 ▸).

A second dataset was collected in SPED mode on a newly prepared grid using a precession semiangle of 0.45°. The acquisition was performed under similar conditions and with the same frame selection criteria as used for the SED dataset, without any rotation of the stage. When the data acquisition was interrupted the next day, a total of 3949 frames (∼2 GB) were retained for subsequent analysis.

### 3.2. Data processing of serial electron diffraction data

The processing of SerialED data can be performed using many of the software tools developed for serial X-ray crystallography, provided that the diffraction geometry is appropriately adjusted. Among the available software packages, *CrystFEL* (White *et al.*, 2012 ▸) stands out as the most widely used for processing serial crystallography data. *CrystFEL* includes many modules for indexing, such as *pinkIndexer* (Gevorkov *et al.*, 2020 ▸), which has been successfully tested on electron diffraction data. A comprehensive guide on data processing of SerialED data using *CrystFEL* and *diffractem* has been provided by Bücker *et al.* (2021 ▸).

In the present study, the different SerialED datasets were processed using *nXDS* (Kabsch, 2014 ▸) and *PETS2* (Palatinus *et al.*, 2019 ▸), with *PETS2* being the only software that can directly process precession electron diffraction data.

#### 3.2.1. Data processing with *nXDS*

*nXDS*, developed by the same author as *XDS* (Kabsch, 2010 ▸), is designed for processing X-ray snapshot data. To the best of our knowledge, *nXDS* has not been explicitly used for the processing of electron diffraction data. The workflow is very similar to that of *XDS*, with the experimental geometry and acquisition parameters specified in the input file nXDS.INP. Because of this close similarity, previous experience using *XDS* will make it easy to set up and run *nXDS*.

During the COLSPOT task, which is responsible for identifying the strongest reflections, various peak-finding parameters are defined within the input file. At this stage, *nXDS* also filters out frames that do not contain a minimum number of reflections, as specified by the keyword MINIMUM_NUMBER_OF_SPOTS. For this dataset, we set this value to 10. As a result, 1029 frames were excluded, leaving us with 25907 frames for subsequent data processing.

The position of the identified spots is then passed to the next task, IDXREF, to determine the orientation and symmetry of the crystal. During this step, we allowed the refinement of the crystal orientation and unit-cell parameters for each frame. A frame was considered successfully indexed if the proportion of indexed reflections, relative to the total number of observed reflections within that frame, was higher than a user-defined threshold (MINIMUM_FRACTION_OF_INDEXED_SPOTS=0.7 in our case). This resulted in 6481 successfully indexed frames, with all other frames being discarded.

The following step is the INTEGRATE task, which calculates the integrated intensities for the indexed reflections. This step refines the reflection profiles and evaluates the quality of the integration process. To ensure accurate measurements, partially integrated reflections are ignored unless at least 75% of their expected intensity has been observed. This threshold is defined in the input file as MINPK=75, allowing *nXDS* to estimate the missing intensity by fitting the learned profiles when this condition is met. Finally, the CORRECT task is executed. In this step, the indexed frames are first reindexed to a common unit-cell basis using the symmetry constraints defined in the input file. The integrated intensities are then corrected and scaled, and their uncertainties estimated. A total of 2335 frames were successfully reindexed, and the resulting dataset statistics are summarized in Table S3.

#### 3.2.2. Data processing with *PETS2*

The data processing procedure used in *PETS2* is as follows. Once the diffraction peaks are identified within each frame during the *Peak Search* step, the *Serial ED* task can be executed. Based on the unit cell and the Laue class supplied, templates with expected peak positions for a set of discrete orientations are generated. These templates are then compared with the experimental frames in order to determine their respective orientations.

While this approach proved to be robust in many cases, visual inspection revealed a significant number of frames where the indexing was unsuccessful. At the time of writing, *PETS2* lacks an automatic mechanism for excluding wrongly indexed frames, which should instead be manually discarded by the user. This manual intervention, while doable for small datasets, would be a very time-consuming process in our analysis, given the size of our datasets.

To address this, we exploited the detailed information provided by *PETS2* within the .dyntmp file. This file, generated during the *Process frames per integration* step, where *PETS2* predicts the positions of reflections on the basis of the orientation matrix, contains crucial parameters for each frame, such as reflection indices with their corresponding intensities, sigmas, observed coordinates, predicted coordinates *etc*. We assumed that frames with incorrect indexing would have a significant discrepancy between the predicted and observed reflection coordinates, resulting in many predicted reflections with very poor *I*/σ ratio. With that in mind, we developed a Python script to filter the frames by retaining only those with a sufficient number of observed peaks exhibiting good agreement with the predicted positions. This filtering process involves the following steps:

(1) Read the .dyntmp file and group the data by frame number.

(2) Discard reflections with a resolution lower than a specified threshold.

(3) Discard reflections with an intensity-to-sigma ratio (*I*/σ) lower than a specified threshold.

(4) Exclude frames with fewer resulting reflections than a specified threshold.

**Figure 6:**
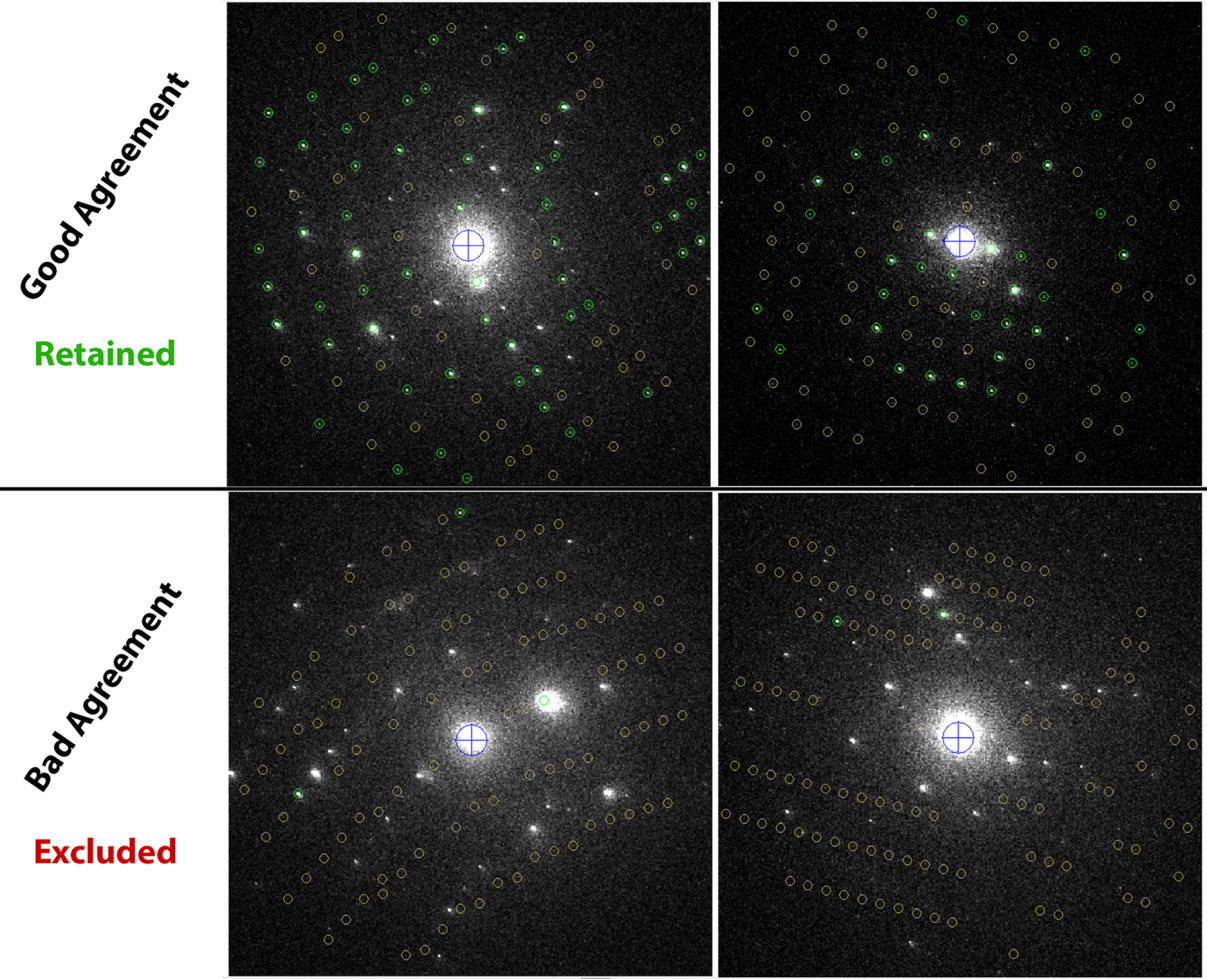
Examples of diffraction images retained (top row) and removed (bottom row) by the Python script. Retained frames show good agreement between predicted and observed reflection positions (green markers), while removed frames have misaligned or insufficient reflections.

In this work, reflections with a resolution lower than 0.1 Å^−1^ or an *I*/σ below 10 were discarded, and frames with fewer than 15 remaining reflections were excluded. The filtered dataset was then reinjected into *PETS2* with the useforcalc flag set to 0 in the input file for all frames that did not meet these criteria, so that they are ignored during data processing. Examples of retained and discarded diffraction patterns are shown in Fig. 6 ▸.

While more sophisticated approaches for discarding unindexed frames may exist, our approach, though potentially susceptible to false negatives (excluding well-indexed frames), effectively removed a large number of unindexed frames that would have significantly degraded the overall quality of the data if not removed. This filtering process resulted in a subset of 4269 (SED) and 2517 (SPED) frames that met the defined criteria.

At this point, the data were processed as static and precession tilt series for SED and SPED datasets, respectively, without using any smoothing method during the geometry optimization steps and with no interframe correlation during the scaling step.

The statistics reported upon running the *Finalize Integration* task are shown in Table S1 and Table S2.

## 4. Results and discussion

**Figure 7:**
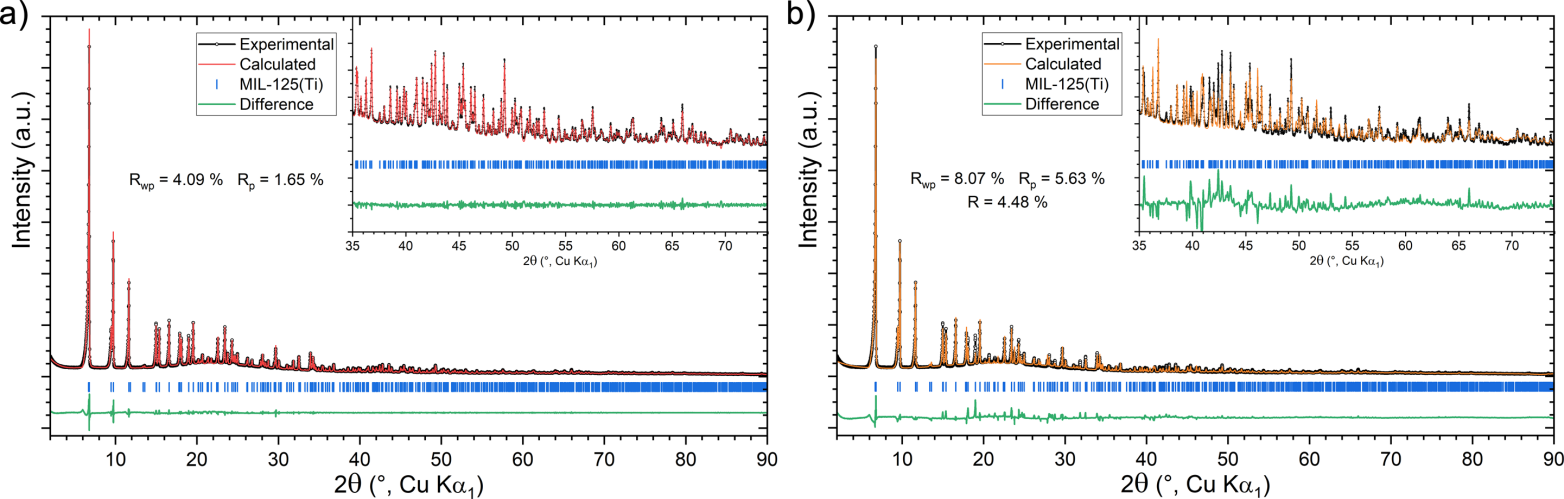
Profile fit from Pawley refinement (*a*) and Rietveld refinement (*b*) of MIL-125(Ti).

Initially, the MIL-125(Ti) powder sample was characterized with PXRD analysis. Its X-ray powder diffraction pattern was indexed with *TOPAS* (Coelho, 2003 ▸; Coelho, 2018 ▸) as tetragonal with cell parameters of *a* = *b* = 18.71 Å and *c* = 18.18 Å. The profile fitting was initially conducted using Pawley refinement, the background was fitted as a Chebychev polynomial, and the peak profile was modelled as a pseudo-Voigt function corrected for axial divergence asymmetry. The refinement converged to *R*_wp_ = 4.09% and *R*_p_ = 1.65%, leading to unit-cell parameters of *a* = *b* = 18.671 (1) Å and *c* = 18.142 (1) Å [Fig. 7 ▸(*a*)]. The unit-cell parameters obtained from PXRD were later used during the indexing step of the SerialED data processing.

**Figure 8:**
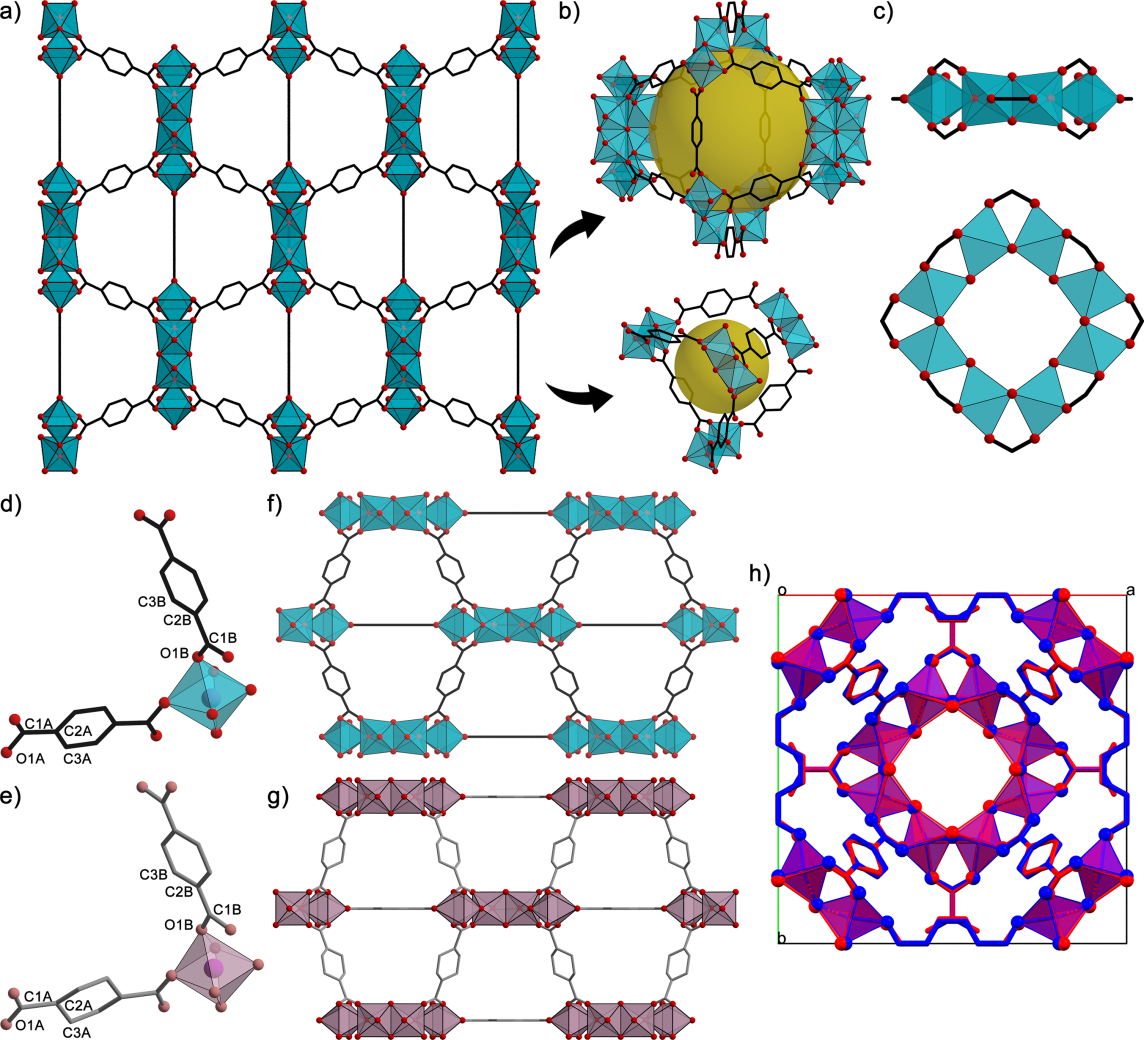
Schematic representation of the MIL-125(Ti) framework obtained from SPED and oriented along the crystallographic *a* axis (*a*). Overview of the octahedral and tetrahedral cavities (*b*) alongside the SBU composing the network (*c*). Comparison of fraction of the polymeric repeating unit (*d*, *e*), the crystal packing oriented along the crystallographic *b* axis (*f*, *g*) and the structure overlay oriented along the crystallographic *c* axis (*h*) between the crystal structure obtained from serial ED (*d*, *f*) and the reported structure from PXRD analysis (*e.g.* Dan-Hardi *et al.*, 2009 ▸). In the structure overlay, the serial ED structure is represented in blue while the PXRD structure is in red. Carbon atoms are represented as black sticks, oxygen atoms as red spheres and Ti(IV) centres as blue polyhedra, while hydrogen atoms have been omitted for clarity.

The structure determination of MIL-125(Ti) was performed using the *SHELXT* package (Sheldrick, 2015*b* ▸), delivering a structural model in space group *I*4/*mmm* on both SED and SPED datasets, with the unit-cell parameters as the only prior crystallographic information. All refinements were performed using the kinematical approximation. Dynamical refinement could in principle be applied to the SPED dataset but would ideally require refining individual crystal thicknesses for each diffraction pattern, significantly increasing both the number of parameters and the computational cost. The kinematical approximation consists of neglecting dynamical scattering and assuming that *I*_*hkl*_ is proportional to |*F*_*hkl*_|^2^. Least-squares structure refinement was performed with the software *SHELXL-2018* (Sheldrick, 2015*a* ▸) interfaced with the *Olex2-1.5* suite (Dolomanov *et al.*, 2009 ▸). The hydrogen atoms were included in the refinement as riding atoms with an idealized geometry. The crystal structure presents the expected MIL-125(Ti) framework [Fig. 8 ▸(*a*)], composed of octahedral and tetrahedral cavities [Fig. 8 ▸(*b*)] connected through octagonal secondary building units [SBUs, Fig. 8 ▸(*c*)]. To have an estimate of the water content, initially, the disordered molecules in the channel were modelled as partially occupied water molecules. However, given that the water content in the channels varies among individual crystals and the data were obtained from thousands of them, a solvent mask was applied using the solvent masking procedure implemented in *Olex2* (Dolomanov *et al.*, 2009 ▸) for the different structures. The use of the solvent mask resulted in a significant reduction of the *R* value (Table 2 ▸) and enabled the estimation of the number of water molecules within the pores, which accounts for 31, 57 and 29 electrons for SED (*PETS2*), SED (*nXDS*) and SPED, respectively (Table 3 ▸). The estimated water content in the asymmetric unit is 3 and 5.5 molecules, according to the 3D-ED models obtained from *PETS2* and *nXDS* data analysis, respectively. The discrepancy highlights the sensitivity of disordered solvent modelling in SerialED and reflects the strong influence of pattern selection, indexing and data quality on such fine structural details.

Moreover, the structures obtained from SED and SPED have been compared through the *Structure Overlay* function implemented in the *Mercury* software (Macrae *et al.*, 2020 ▸), revealing a root-mean-square value of 0.035 (Fig. S5). The agreement between the structural models highlights the reproducibility of these data collection protocols, with no significant deviation in terms of atomic coordinates or residual potential within the pores. Data processing and refinement statistics of the different models are reported in Table 4 ▸.

A comparison between the MIL-125(Ti) structures obtained here and the model reported in the Cambridge Structural Database (CSD), derived from PXRD data, shows that the SerialED models provide a more accurate representation of the organic moieties. Specifically, the structure obtained from SPED exhibits all the phenyl rings of the linkers in a nearly planar geometry [Figs. 8 ▸(*d*)–8 ▸(*g*)]. Furthermore, we compared the bond lengths of the organic fragments in the different models with CSD average values obtained from 3984 terephthalate-containing structures. The comparison with the crystallographic database highlights a higher accuracy in the bond length for the structures reported in this work (Table S4). Subsequently, a Rietveld refinement was performed starting from the atomic coordinates obtained by the SerialED model. This refinement was conducted by considering the polymeric backbone as rigid and refining the residual density in the channels as disordered water molecules. The residual water modelled in the pores accounts for 5 water molecules, which is in line with the number estimated through thermogravimetric analysis(TGA; Fig. S3). The refinement converged to *R*_wp_ = 8.07%, *R*_p_ = 5.63% and *R* = 4.48%, leading to unit-cell parameters of *a* = *b* = 18.6777 (2) Å and *c* = 18.1470 (3) Å [Fig. 7 ▸(*b*)]. The SerialED method provides a structural model that is representative of the entire sample, as it is derived from thousands of diffraction patterns from different crystals, and this is further supported by the consistency observed in the PXRD refinement. The only remarkable difference is the solvent content estimate within the pores from the different serialED models. Unfortunately, the PXRD data cannot be used to determine which of the different SerialED models is more accurate as the vacuum conditions inside the TEM column may reduce the amount of water present in the channels [Fig. 7 ▸(*b*)].

## 5. Conclusions

In this work, we have described the implementation of SerialED by stage scanning within *LibraEDT*, enabling largely unsupervised data acquisition through controlled stage movement, real-time peak evaluation and flexible filtering criteria. This approach directly addresses one of the main practical limitations that has so far restricted the wider adoption of SerialED while also allowing the use of the microscope outside standard working hours. In fact, all datasets presented in this study were collected overnight, during periods when the instrument would otherwise have remained idle. While the current implementation targets the Zeiss Libra 120, the approach can be adapted to any TEM providing an API for stage control and a detector API supporting acquisition at different frame rates.

We then showed how the datasets collected with and without precession, referred to as SPED and SED, can be processed using *PETS2* and *nXDS* with very little additional effort. This makes the data processing step, which is often considered one of the most challenging and time-consuming parts of SerialED, much easier and more similar to the standard procedures used for conventional 3D-ED.

Finally, the successful structural analysis of the beam-sensitive MOF MIL-125(Ti) demonstrates the reliability and practicality of this SerialED implementation. The good agreement obtained for both SED and SPED datasets confirms the robustness of the method and highlights its potential for the structural investigation of materials that are otherwise difficult to analyse using conventional electron diffraction approaches.

## Supplementary Material

Crystal structure: contains datablock(s) global, mil_125_sed_pets2, mil_125_sped_pets2, mil_125_sed_nxds. DOI: 10.1107/S1600576726003894/te5163sup1.cif

Supporting information file. DOI: 10.1107/S1600576726003894/te5163sup2.pdf

CCDC references: 2518470, 2518471, 2518472

## Figures and Tables

**Table:** Common data acquisition parameters (TEM and STEM).

Data acquisition software	*LibraEDT*
Data acquisition mode	Stage movement
Beam size	≈ 300 nm
Stage movement speed	10 µm s^−1^
Exposure time	50 ms per frame
Frame coverage	0.5 µm per frame
Electron fluence per crystal	≈ 12 e^−^ Å^−2^
Acquisition duration	≈ 15 h
Total images captured	Over 1000000
Filtering conditions	At least 5 peaks with *I*/σ > 5 AND resolution better than 1.25 Å

**Table:** Differing parameters.

Parameter	SED	SPED
Illumination mode	TEM	STEM
Precession angle	None	0.45°
Precession cycles per frame	None	5
Random stage rotation	Every 45 min	None
Retained frames	26962	3949

**Table 2:** Refinement parameter comparison as a function of solvent mask usage

	SED (*nXDS*)		SED (*PETS2*)		SPED	
	No mask	Mask	No mask	Mask	No mask	Mask
*R*_1_(obs)	0.3206	0.1935	0.3278	0.1890	0.3143	0.1878
*wR*_2_(obs)	0.5985	0.4289	0.6431	0.4473	0.6639	0.4540
*R*_1_(all)	0.3372	0.1983	0.3347	0.1917	0.3169	0.1891
*wR*_2_(all)	0.6074	0.4321	0.6446	0.4502	0.6649	0.4553
Max. peak (e^−^ Å^−3^)	0.49	0.19	0.29	0.23	0.25	0.43
Min. peak (e^−^ Å^−3^)	−0.57	−0.50	−0.42	−0.32	−0.55	−0.31

**Table 3:** Water content per asymmetric unit estimation from TGA, PXRD, SED and SPED analysis

Technique	Water molecules
TGA	8
PXRD	5
SED (*nXDS*)	5.5
SED (*PETS2*)	3
SPED	3

**Table 4:** Crystallographic information for MIL-125(Ti) from SED and SPED analysis

	SED (*nXDS*)	SED (*PETS2*)	SPED
Empirical formula	C_12_H_6_O_9_Ti_2_	C_12_H_6_O_9_Ti_2_	C_12_H_6_O_9_Ti_2_
Formula weight	389.91	389.91	389.91
Temperature (K)	293 (2)	293 (2)	293 (2)
Crystal system	Tetragonal	Tetragonal	Tetragonal
Space group	*I*4/*mmm*	*I*4/*mmm*	*I*4/*mmm*
*a*, *b* (Å)	18.740 (3)	18.7996 (4)	18.634 (1)
*c* (Å)	18.220 (4)	18.4774 (6)	18.255 (2)
Volume (Å^3^)	6399 (2)	6530.4 (3)	6338.6 (7)
*Z*	8	8	8
ρ_calc_ (g cm^−3^)	0.809	0.793	0.817
2θ range (°)	0.144 to 1.908	0.288 to 1.92	0.146 to 1.916
Reflections collected	1928	10705	10745
Independent reflections	965 (*R*_int_ = 0.1034, *R*_σ_ = 0.1411)	1007 (*R*_int_ = 0.2617, *R*_σ_ = 0.1305)	979 (*R*_int_ = 0.1849, *R*_σ_ = 0.1108)
Data/restraints/parameters	965/50/33	1007/50/33	979/50/33
Goodness of fit on *F*^2^	1.556	1.131	1.198
Final *R* indexes [*I* ≥ 2σ(*I*)]	*R*_1_ = 0.1935, *wR*_2_ = 0.4289	*R*_1_ = 0.1890, *wR*_2_ = 0.4473	*R*_1_ = 0.1878, *wR*_2_ = 0.4540
Final *R* indexes (all data)	*R*_1_ = 0.1983, *wR*_2_ = 0.4327	*R*_1_ = 0.1917, *wR*_2_ = 0.4502	*R*_1_ = 0.1891, *wR*_2_ = 0.4553
Largest difference peak/hole (e^−^ Å^−3^)	0.19/−0.50	0.23/−0.32	0.43/−0.31

## Data Availability

The source code of the software discussed in this paper is freely available on Github (https://github.com/Thioo/LibraEDT_Timepix_Server and https://github.com/Thioo/Zeiss-Libra-3DED-Data-Acquisition). The crystal structures have been deposited with the Cambridge Crystallographic Data Centre under deposition numbers 2518472 (MIL-125-SED-nXDS), 2518471 (MIL-125-SED-PETS2) and 2518470 (MIL-125-SPED-PETS2).
