# Supplementary material for: Automated serial electron diffraction: implementation in LibraEDT and its applications
Source: J Appl Crystallogr. 2026 May 8;59(Pt 3):858–68. doi: 10.1107/S1600576726003894 (PMC13224799; doi:10.1107/S1600576726003894)
Supplement: Supplementary file 2 [file j-59-00858-sup2.pdf]

JOURNAL OF APPLIED CRYSTALLOGRAPHY

Volume 59 (2026)

Supporting information for article:

Automated serial electron diffraction: implementation in *LibraEDT* and its applications

Moussa D. Faye Diouf, Danilo Marchetti, Paola Parlanti, Alessandro Pedrini and Mauro Gemmi

Supplementary Information

5.1 LibraEDT Software

To support the description of the SerialED implementation, an overview of the LibraEDT graphical user interface is shown in Figure S1. A detailed description of the software architecture is provided in our previous publication (Faye Diouf & Gemmi, 2025).

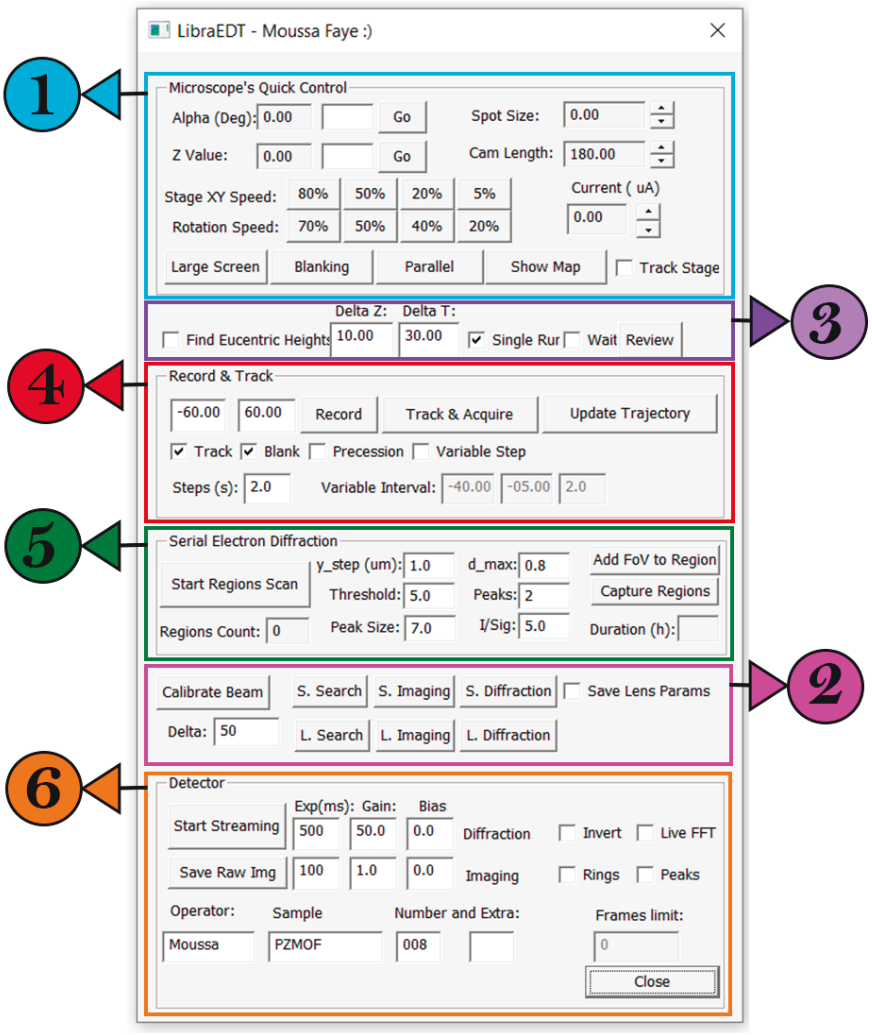

Figure S1: LibraEDT Software Graphical User Interface (GUI). The present work focuses on the implementation of Section 5. The sections were described in detail in our previous publication.

An overview of the interactive 2D map used to set up SerialED scan regions is provided in Figure S2. This interface enables region definition and real-time visualization of the stage position.

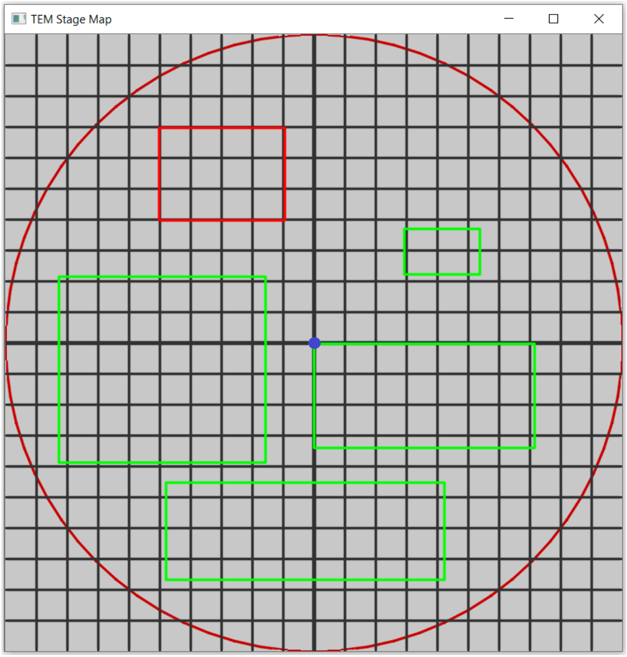

Figure S2: Interactive 2D map interface in LibraEDT for SerialED data acquisition. Users can freely define rectangular regions for scanning. Green regions indicate areas scheduled for acquisition, while red regions correspond to already scanned areas. The blue dot marks the current stage position.

5.2 Material Preparation

5.2.1 Synthesis of MIL-125(Ti)

The synthesis of MIL-125(Ti) was conducted, adapting a previously reported procedure (Sun *et al.*, 2024), by solubilising 1.03 g of terephthalic acid (H_2TA, 6.22 mmol) in 10 mL of DMF, inside a 45 mL glass tube with a crewed tap equipped with a PTFE sealing. Then 520 μL of $Ti(OiPr)_4$ (1.71 mmol) were added dropwise into 2 mL of methanol under stirring, leading to a white suspension. The suspension of Ti(IV) was then added to the H_2TA solution and left under stirring at room temperature for 30 min. The reaction mixture was heated at 130 °C and left under stirring for 24 hours. The product was filtered under reduced pressure and washed with 30 mL of DMF and 40 mL of methanol, subsequently heated under dynamic vacuum ($5 \cdot 10^{-2}$ torr) at 130 °C for 4 hours. The obtained white powder (323 mg) was characterised through powder X-ray diffraction (PXRD), 3D ED, thermogravimetric analysis (TGA) and N_2 adsorption at 77 K.

5.2.2 Thermogravimetric Analysis

The thermogravimetric analysis (TGA) was performed on a Perkin Elmer Instrument, model TGA 8000. The experiments were carried out in the temperature range 30-450 °C with a heating rate of 10 °C min^{-1} under air flow. The recorded thermogram shows a weight loss of 15.5%, which is related to the desorption of water molecules (Figure S3). The presence of water molecules in the pores results from exposure to atmospheric moisture, showing that MIL-125(Ti) readily alter its

water content when in contact with air. Based on the measured weight loss, the water content in MIL-125(Ti) was estimated to be equivalent to about eight water molecules in the asymmetric unit.

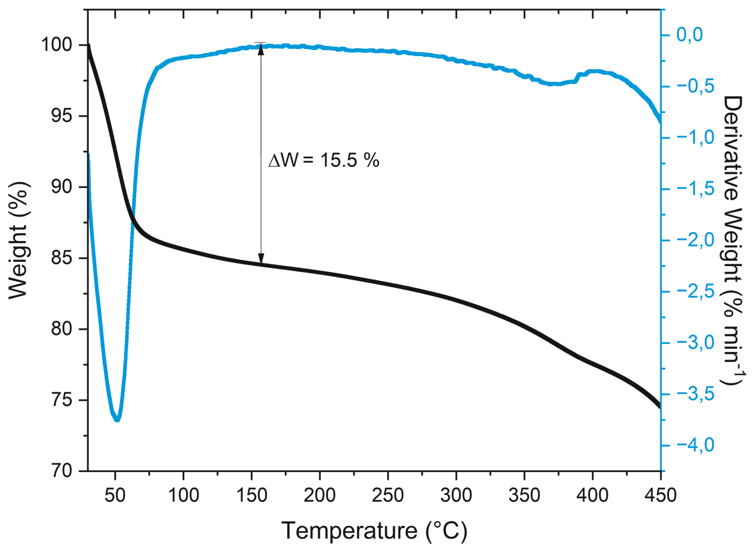

Figure S3: Thermogravimetric path recorded on the MIL-125(Ti) sample.

5.2.3 Nitrogen sorption measurement

Nitrogen adsorption isotherms at 77 K were acquired using a Microtrac Belsorp Max X instrument. The powder was pretreated at 130 °C for 16 hours using a Belprep Vac III station, followed by a subsequent treatment at the analysis port at 130 °C for 90 minutes under pressures below 1 Pa. The specific surface area was calculated using the BET model within a pressure range, identified from the Rouquerol plot, and corresponding to 0.005–0.05 p/p^0. The calculated surface area accounts for 1612 m^2g^{-1}, which is in line with the data reported in the literature (Dan-Hardi *et al.*, 2009; Sun *et al.*, 2024). The pore size distribution was obtained by fitting the adsorption isotherm using the non-local density functional theory (NLDFT) method, assuming a "metal oxide" model with a cylindrical pore geometry (Figure S4). The calculation provides a monomodal distribution with a maximum at 1.2 nm, which is in line with the maximum pore diameter obtained from the structural model.

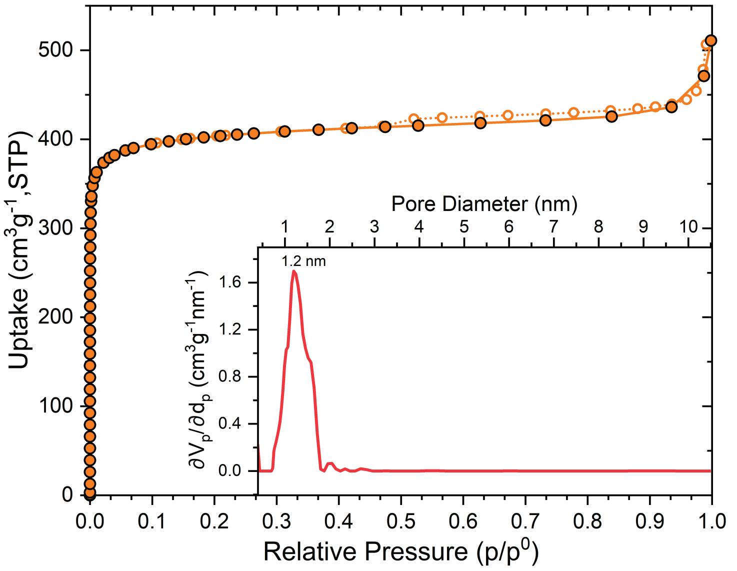

Figure S4: Nitrogen adsorption isotherm of MIL-125(Ti) and its relative pore size distribution.

5.3 Data Processing Statistics

Detailed integration and scaling statistics for each dataset and data processing technique are provided in the tables below. These values serve as a quantitative evaluation of data quality and complement the discussion presented in the main manuscript.

5.3.1 PETS2

Table S1: SED - Integration and Scaling statistics using PETS2. In bold is the resolution cut-off.

d^*-range	d-range	Nobs	Nall	Nthr	compl.	rnd. I/σ(cnt)	I/σ(err)	Rint(obs)	Rint(all)	CC1/2
0.00–0.33	Inf–3.00	38	45	53	0.849	8.67	66.32	4.60	12.58	97.21
0.33–0.47	3.00–2.12	50	75	75	1.000	9.60	47.86	3.66	17.53	97.49
0.47–0.58	2.12–1.73	38	88	92	0.957	10.55	35.30	3.11	18.28	96.46
0.58–0.67	1.73–1.50	17	105	107	0.981	10.73	19.11	2.11	18.03	94.01
0.67–0.75	1.50–1.34	17	106	114	0.930	11.38	18.57	2.19	19.87	94.25
0.75–0.82	1.34–1.22	11	124	131	0.947	11.40	12.24	1.65	22.40	77.61
0.82–0.88	1.22–1.13	4	140	141	0.993	11.11	10.24	1.69	20.22	90.93
0.88–0.94	1.13–1.06	3	145	145	1.000	11.13	8.53	1.36	27.07	74.94
0.94–1.00	1.06–1.00	1	154	155	0.994	11.38	7.01	1.17	28.90	73.45
**0.00–1.00**	**Inf–1.00**	**179**	**982**	**1013**	**0.969**	**10.90**	**22.54**	**2.57**	**16.36**	**97.88**

Table S2: SPED - Integration and Scaling statistics using PETS2. In bold is the resolution cut-off.

d^*-range	d-range	Nobs	Nall	Nthr	compl.	rnd. I/σ(cnt)	I/σ(err)	Rint(obs)	Rint(all)	CC1/2
0.00–0.33	Inf–3.00	44	46	51	0.902	8.61	29.85	6.61	9.61	96.88
0.33–0.47	3.00–2.12	59	70	75	0.933	10.11	13.35	5.11	23.65	85.76
0.47–0.58	2.12–1.73	53	82	86	0.953	10.66	9.10	4.28	17.64	97.74
0.58–0.67	1.73–1.50	51	101	104	0.971	11.13	4.90	2.91	21.26	94.43
0.67–0.75	1.50–1.34	48	104	109	0.954	11.60	3.51	2.75	21.30	90.42
0.75–0.82	1.34–1.23	34	125	125	1.000	11.22	2.88	2.55	20.49	95.25
0.82–0.88	1.23–1.13	22	136	136	1.000	11.53	2.07	2.15	21.98	94.21
0.88–0.94	1.13–1.06	10	146	146	1.000	11.73	1.49	1.61	24.00	81.64
0.94–1.00	1.06–1.00	7	147	147	1.000	11.93	1.31	1.55	21.32	85.72
**0.00–1.00**	**Inf–1.00**	**328**	**957**	**979**	**0.978**	**11.23**	**5.88**	**3.80**	**15.84**	**95.00**

5.3.2 nXDS

Table S3: SED - Integration and Scaling statistics using nXDS. In bold is the resolution cut-off.

RESLIM	OBSERVED	UNIQUE	POSSIBLE	COMPLETE	I/SIGMA	CHI-test	R_{mrgd}-F	CC(1/2)
5.160	3224	12	12	100.0	22.5	1.558	6.1	93.5*
3.779	3828	15	15	100.0	18.2	1.459	7.6	93.0*
3.123	4787	19	19	100.0	14.6	0.954	4.8	96.3*
2.722	4063	17	17	100.0	12.6	0.994	6.0	98.7*
2.443	5311	24	25	96.0	11.8	1.288	9.0	97.7*
2.236	4438	21	21	100.0	9.8	0.669	4.8	99.5*
2.074	4917	25	25	100.0	12.0	1.165	8.3	93.6*
1.943	4592	23	24	95.8	10.8	0.783	5.7	98.6*
1.834	4733	28	28	100.0	7.5	0.787	9.2	97.5*
1.741	4361	29	29	100.0	7.9	0.720	7.2	99.7*
1.661	4283	30	30	100.0	6.7	0.786	10.6	98.2*
1.591	3470	30	30	100.0	5.8	0.692	11.2	97.9*
1.530	3899	33	33	100.0	6.2	0.738	10.0	96.0*
1.475	2767	28	28	100.0	5.6	1.066	16.6	88.4*
1.425	4090	40	40	100.0	7.8	1.063	11.6	95.0*
1.380	2910	32	32	100.0	7.0	1.046	15.0	96.2*
1.339	2295	32	32	100.0	5.4	0.766	12.4	97.8*
1.302	2707	39	39	100.0	5.7	1.128	18.1	93.9*
1.268	2034	36	36	100.0	5.1	0.837	15.2	92.7*
1.236	2071	39	39	100.0	5.5	0.958	16.7	95.2*
1.206	2127	40	40	100.0	7.1	1.396	18.4	92.0*
1.179	1550	39	39	100.0	3.9	1.144	28.9	70.5*
1.153	1316	39	39	100.0	3.7	0.950	25.8	84.6*
1.129	1451	47	47	100.0	4.1	0.994	24.1	86.1*
1.106	1042	39	39	100.0	3.8	1.083	29.8	91.8*
1.085	779	39	39	100.0	3.4	1.046	36.6	71.4*
1.065	735	42	42	100.0	4.1	1.144	33.4	73.0*
1.046	606	45	45	100.0	3.1	0.972	33.7	47.0
1.028	524	46	46	100.0	2.8	1.097	45.0	42.6
**1.010**	**221**	**37**	**42**	**88.1**	**2.4**	**1.242**	**73.9**	**-17.3**

5.4 Structural Comparisons

5.4.1 Bond Distances Comparison

Table S4: Bond distance comparison between the crystal structure obtained from serial ED, the structure reported in literature from PXRD analysis (Dan-Hardi *et al.*, 2009), and the mean values for similar bonds in the structures reported in the CSD.

Atom 1	Symmetry 1	Atom 2	Symmetry 2	SED (PETS2)	PXRD ref	CSD
C1A	x, y, z	O1A	x, y, z	1.308(10)	1.18943(9)	1.256(30)
C1A	x, y, z	C2A	x, y, z	1.480(12)	1.6573(2)	1.502(20)
C2A	x, y, z	C3A	x, y, z	1.387(5)	1.3952(1)	1.386(17)
C3A	x, y, z	C3A	-1/2+y, 1/2+x, 3/2-z	1.391(7)	1.4726(2)	1.385(18)
C1B	x, y, z	O1B	x, y, z	1.281(14)	1.3101(11)	1.256(30)
C1B	x, y, z	C2B	x, y, z	1.501(15)	1.4140(2)	1.502(20)
C2B	x, y, z	C3B	x, y, z	1.391(5)	1.4826(1)	1.386(17)
C3B	x, y, z	C3B	x, 2-y, 1-z	1.390(7)	1.3729(2)	1.385(18)

5.4.2 Asymmetric unit comparison

A comparison of the asymmetric units obtained from the SED and SPED datasets is presented in Figure S5, highlighting the high degree of structural agreement between the two models.

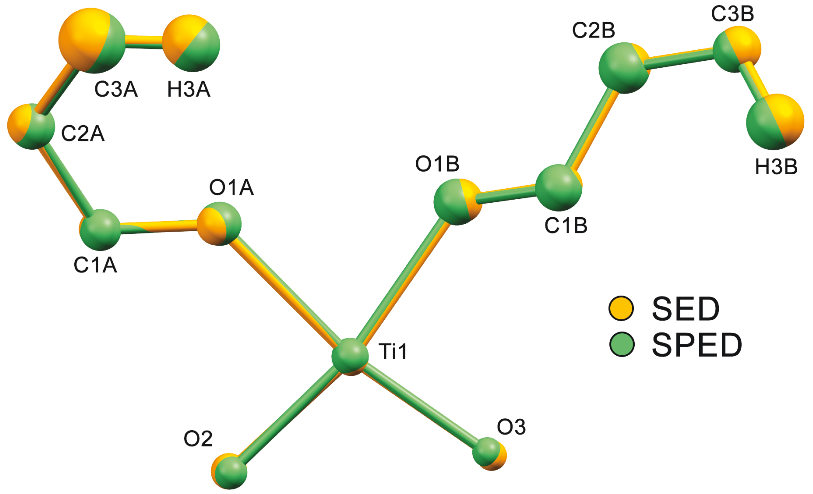

Figure S5: Comparison between the ORTEP view of the asymmetric unit of MIL-125(Ti) structures obtained from SED and SPED data collection (probability level 30 %).

References

Faye Diouf, M. D. & Gemmi, M. (2025). *Journal of Applied Crystallography*, **58**(5). https://doi.org/10.1107/S1600576725006892

Dan-Hardi, M., Serre, C., Frot, T., Rozes, L., Maurin, G., Sanchez, C. & Ferey, G. (2009). *Journal of the American Chemical Society*, **131**(31), 10857–10859.

Sun, Y., Ji, H., Sun, Y., Zhang, G., Zhou, H., Cao, S., Liu, S., Zhang, L., Li, W., Zhu, X. & Pang, H. (2024). *Angewandte Chemie International Edition*, **63**(3), e202316973.
